# Yet Another Non-Unique Human Behaviour: Leave-Taking in Wild Chacma Baboons (*Papio ursinus*)

**DOI:** 10.3390/ani12192577

**Published:** 2022-09-27

**Authors:** Lucy Baehren, Susana Carvalho

**Affiliations:** 1 Primate Models for Behavioural Evolution Lab, Institute of Human Sciences, University of Oxford, Oxford OX2 6PR, UK; 2 Gorongosa National Park, Beira 1983, Mozambique; 3 Interdisciplinary Centre for Archaeology and Evolution of Human Behaviour, Universidade do Algarve, 8005-139 Faro, Portugal

**Keywords:** *Papio ursinus*, greeting, leave taking, separation, parting

## Abstract

**Simple Summary:**

Although greeting is well-studied across animal species, its counterpart, leave taking, is little studied in nonhumans. Here, we review the previous limitations of leave-taking research and use this to develop a new method for studying leave taking in nonhumans. Using videos of chacma baboons in Gorongosa National Park, Mozambique, we compared behaviours at the end of social departures to nonsocial departures. We found that shifting orientation towards the direction of parting was significantly more likely in social departures compared to nonsocial departures. As the first evidence of leave taking in a wild nonhuman species, we suggest that leave taking is not uniquely human as previously argued, and that our method could be used to further explore the presence of leave taking in other nonhuman species.

**Abstract:**

Leave taking is a common, possibly universal, feature of human social behaviour that has undergone very little empirical research. Although the importance remains unknown, it has been suggested to play an important role in managing separations, mitigating the risk, and increasing social bonding beyond the interaction itself. In nonhuman species, the literature is virtually absent, but identifying leave taking beyond humans may provide unique insights into the evolutionary history of this behaviour and shed light onto its proximate and ultimate function(s). Methods to study leave taking are not well-established, and the variation in definitions, measures, and control variables presented in past studies poses additional challenges. Baboons are a valuable model for investigating human behavioural evolution: as a flexible, highly adaptable, and social primate whose radiation is, similarly to humans, associated with the emergence of the African savannah biome. Using the framework and definition proposed by Baehren, we investigated the presence of leave taking in a wild, generalist primate and tested a range of candidate behaviours on prerecorded video footage: (1) self-scratching, (2) eye gaze, and (3) orientation in the direction of parting. Using multivariate analysis, controlling for interaction duration and individual variation, our results show that orientation in the direction of parting occurs predominantly before social separation events. These results indicate evidence of leave taking in a wild nonhuman population and contrast with previous ideas that this is a uniquely human behaviour. The presence of leave taking in baboons suggests a deep evolutionary history of this behaviour, warranting further investigation into its function and presence across other nonhuman primate species.

## 1. Introduction

### 1.1. Lack of Leave-Taking Study

Compared to the study of greeting, the ways in which interactions end are much less well-studied. This difference is exacerbated when looking at nonhuman species [1]. Nonhuman primates provide useful models for understanding how behaviour has evolved in humans, or how adaptive behaviours have converged [2]. For example, studies of greeting in chimpanzees suggest that these behaviours function to reduce tension and increase social bonding upon meeting [3]. The presence of greeting in nonhuman species suggests that this behaviour has an ancient origin that precedes the emergence of *Homo sapiens*, or in fact, the entire primate Order [4]. Indeed, Jane Goodall claimed that some of the most convincing similarities between animal and human studies are shown in greeting behaviour [5]. In contrast to this, studies of leave taking in nonhuman species are largely absent, and the origins in our lineage remain unknown. 

The reason for this discrepancy in nonhumans remains unclear, as the behaviours have typically been considered together in humans. A handful of suggestions have been made as to why leave taking has not ‘taken off’ in the same way as greeting: it is hard to measure in animals, requiring more advanced technology to capture retrospective behaviours [6] or it may simply not exist [7]. However, other sources suggest that there may be some evidence of nonhuman leave taking, even if it is does not parallel human leave taking as greetings often do [8]. There are anecdotal reports of primate species showing possible leave-taking behaviours (e.g., [9]), although these reports have never been subject to empirical study. 

Furthermore, although the studies on human leave taking are greater in number, they mostly concern the linguistic structure of conversational endings [1]. Although these methods are appropriate for answering questions of conversation and language, they are less useful for tackling questions of evolution, especially across species. A potentially more useful avenue is to consider nonverbal correlates of parting, across cultures and species. This is useful in two ways: (1) behaviours independent from articulated language may show continuity across species and cultures, and thus inform us of similarities and differences [10]; and (2) may reflect basal aspects of shared communication across primates, as with greeting behaviours [8]. It has been suggested that leave taking should be studied in nonhuman species, for example, where postural signals may serve an analogous function [11], or when considering studies of group movement [12]. Ethological studies of leave taking are lacking, and those that do exist (e.g., [11,13]) are not robust in providing replicable methods. Below, we consider and address possible ways to overcome these limitations. 

### 1.2. Addressing Previous Limitations

In the review from Baehren [1], there is a thorough discussion of the ways in which definitions of leave taking vary. This is a significant methodological issue, in part because it makes studies impossible to compare or reproduce, as the phenomenon of leave taking varies. Almost all definitions relate leave taking to breaking contact, and many seem to also include breaking “social” contact, i.e., that moving apart is not enough; rather, it involves the ending of interaction. In short, significant variation in leave-taking definitions exists, with many studies omitting an operational definition entirely, making it difficult to compare studies. Additionally, Baehren [1] also discusses the need to define the level of study, i.e., what conceptualisation of separation is used. This varies considerably across the existing studies and makes it hard to ensure similar phenomena are being compared. Can we include the everyday comings and goings and ends of conversations, even when individuals remain in the same place (e.g., [13])? Can we only include departures which mark a complete removal from sight and contact (e.g., [4])? We argue that these levels of separation should be considered and acknowledged, including the everyday separations in nonhuman species. One reason for the shortage of previous studies could be the lack of appropriate data collection methods for leave taking: a necessarily difficult phenomenon to study due to the retrospective nature of it, i.e., the behaviour occurs before the visible separation. This point is raised by Albert and Kessler [6], who claim that the properties of leave taking make it difficult to study, in part due to the shortness and high-speed nature of the behaviour. Their suggestion that leave taking could only be properly studied once technology allowed a permanent record to be created and retrospectively examined seems to have now been realised. Several studies attempt this in humans (e.g., [13,14,15]) but there is only one example of this being applied to nonhuman species, in the grey mouse lemur [4]. As suggested by Baehren [1], these advances of both video technology and video-coding software could provide promising solutions to increase the availability of data on leave taking. Whittaker et al. [13] measured human verbal cues and orientating signs in closing sequences, described as “turning away”. We thus include nonvocal, orientating behaviours that may be relevant to interaction endings. To ensure that behaviours measured are unique to social interaction endings, we compare to nonsocial endings. In a study by Grafenhain et al. [15], children were found to show leave taking more frequently in the context of joint activity, as opposed to leaving a solo activity in close proximity to another individual. This suggests that the social nature of the interaction is, at least in part, driving the leave taking. Besides the need to control for social interaction, many studies neglect controlling for the length of interaction. Lockard [11] includes length of interaction as a variable, which failed to reach significance, concluding that restlessness or boredom alone is not sufficient in explaining these behaviours; however, this possibility is still important to account for. 

### 1.3. Baboon Models for Leave Taking

Baboons share a common ancestor with humans from between 21 and 25 million years ago [16,17]. Broadly speaking, several baboon species are grouped together because of similar ecology and social organisation and are described as savannah baboons [18]. These species include yellow baboons (*Papio cynocephalus*), chacma baboons (*Papio ursinus*), and olive baboons (*Papio anubis*). Savannah baboons live in large, cohesive, multimale, multifemale, and female-bonded groups where the males tend to disperse out of their natal group and the females are philopatric [19]. Savannah baboons inhabit mosaic savannah and woodland environments like the Pleistocene environments where our genus emerged [2]. Marked interspecies variation in baboon social organisation is particularly interesting and, as such, they are one of the most studied primate species [20]. Although baboon social systems have historically been thought to be a dichotomy, between the multilevel hamadryas and the cohesive savannah baboons, many argue that we must allow for greater variability in our understanding [20]. This variation would also allow greater understanding of the typically cohesive species, which are usually stable, but do split up on occasion, e.g., when foraging in habitats with scarcer resources or greater predation pressure. 

In several ways, savannah baboons are useful for thinking about the selective pressures that sociality has placed on social structure and the resultant interactions. For example, bonds between females, particularly kin, are thought to be especially important in savannah baboons, who form strong, equitable, enduring bonds with specific female partners [21]. These bonds are adaptive and have a direct effect on fitness, as shown by the positive association with infant survival [22] as well as adult female lifespan [21]. When interacting with other nonrelated individuals, they demonstrate a form of “emotional bookkeeping”, influenced by previous affiliative interactions and forming the strongest bonds with those with whom they have the most balanced and reciprocal grooming interactions [23]. So called “friendships” between male and females, beyond mating, are particularly well-modelled in baboons [24]. Male mammals are expected to care for offspring only when it is directed towards their related young [25], but in cercopithecines, this seems to be the case even with low paternity certainty [26]. This is thought to play a role as a counter strategy to infanticide in chacma baboons, but in more tolerant species where infanticide is rare, it may provide protection against nonlethal forms of aggression [27]. Similarly, in yellow baboons, mothers are found to benefit directly from these friendships, gaining more protection from harassment, usually from genetic fathers [28]. Furthermore, baboons have been touted as a valuable model for the evolution of human language due to their domain-general cognitive functions and gestural production [29]. In contrast to traditional ideas that human language arose due to uniquely human changes (e.g., [30]), new perspectives suggest that language evolved from a combination of components which can be investigated in other living primate species, of which baboons provide a relevant example due to the diversity of research available [29]. Within their communicative behaviour, baboons are known to coordinate group movement [31,32] and display ritualized greeting when they come together [18]. Furthermore, evolutionary continuity between baboons and great apes, as well as similar neural specialization, suggests that such gestural communication could have an ancient Catarrhine origin 25–40 million years ago [33]. In short, savannah baboons provide promising close-knit social groups to investigate short-term departures within cohesive groups, and are a good model for investigating the origins of potential leave-taking behaviours. The presence of such behaviour in baboons would be significant, implying a deeper evolutionary history of the behaviour or convergent evolution to meet a similar socioecological need. 

In this study, we present a new framework for studying leave taking in nonhuman primates, using chacma baboons *Papio ursinus* as an example. We first test the definition and framework for leave taking presented in Baehren’s work [1]. Additionally, we determine which behaviours are useful as candidate leave-taking behaviours. We then employ the use of new data collection methods to collect and control for behavioural patterns, determining whether this framework can highlight the importance of social interaction in leave taking, and allow more nonhuman species to be studied. We predict that, based on the social definition [1], leave taking is significantly more likely in social separation compared to proximity or solo departure. Finally, we discuss the results in an evolutionary context, highlighting the advantages of applying our approach to further studies of nonhuman primates. 

## 2. Materials and Methods

### 2.1. Addressing Methodological Issues

#### 2.1.1. Using a Considered Definition

Here, we used the definition “behaviours occurring before separation from social interaction” to refer to leave taking, as outlined by Baehren [1]. To this end, we focused on the sociality of interaction as a necessary precursor to leave taking, which allowed us to differentiate between behaviours that just occur before movement versus the socially charged ending of an encounter. 

#### 2.1.2. Defining the Level of Study

**Figure 1:**
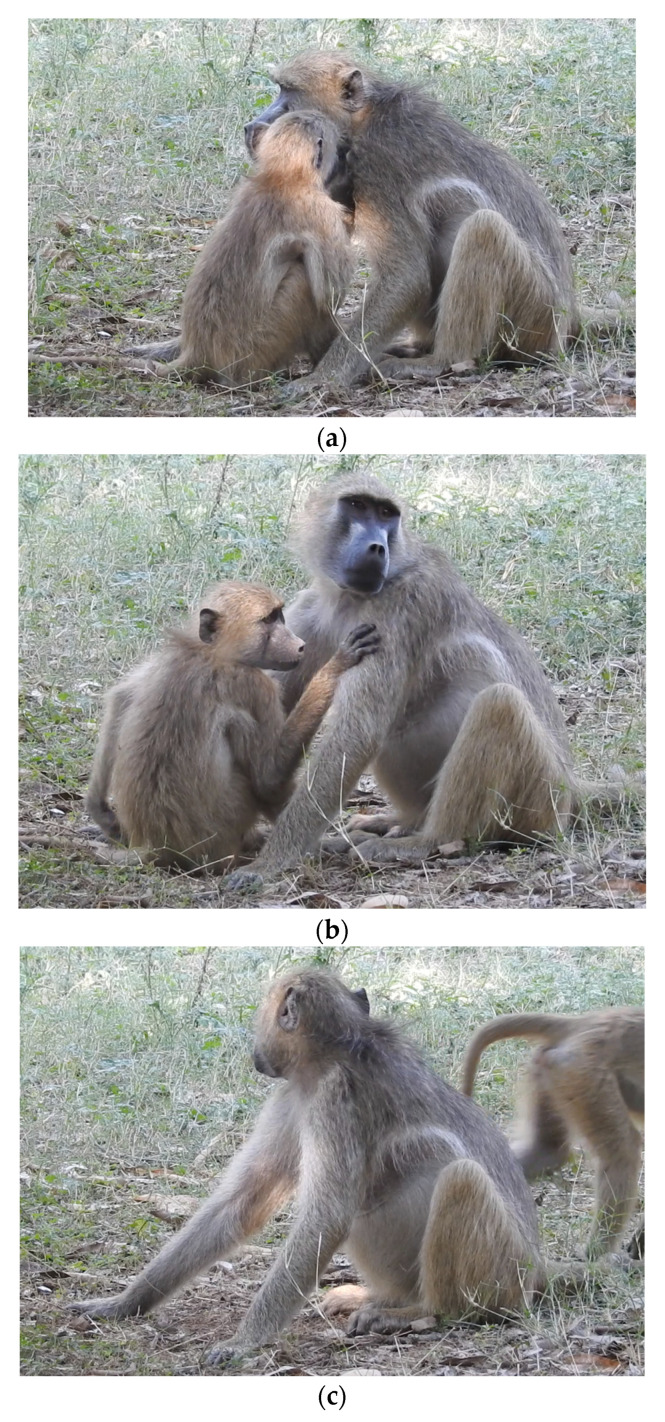
(**a**): An activity is occurring between a dyad. (**b**): The preparting window two minutes before departure; individuals often stop the activity and may perform “leave-taking” behaviours. (**c**): An individual departs, moving away from the other individual in the dyad by >1 m.

In Baehren’s review [1], the level of study is discussed as being an area of particular confusion across studies of leave taking. In the daily following of free-ranging wild populations, it is often not possible to see great numbers of long-term separations, e.g., group departure, transfer, etc. These events are relatively infrequent and would not yield high amounts of data to investigate the associated behaviours. However, in cohesive groups, it should be possible to look at the endings of daily interactions that happen frequently between individuals of the same social group. Although these interactions may not have the “finality” of longer separations, the frequency of their occurrence and their potential function in the ongoing social relationships within the group might mean that they are in fact particularly important for our understanding of leave taking. In this study, we looked at the low-level leave takings, endings of social interactions within a cohesive group of chacma baboons (Figure 1a–c). This allowed the practical benefit of an increased sample size of separation events, but also the detailed investigation of these “every day” social separations from dyad interactions.

#### 2.1.3. Data Collection Methods

Video footage was taken during daily following on a Nikon P900 83X Optical Zoom (Nikon, Tokyo, Japan) mounted on a tripod. This was carried out in an opportunistic way from when at least one individual was visible until all the group had moved out of the shot, thus maximising footage of spontaneous interaction, solo behaviours, and parting events. Details of the footage can be found in the results. The footage was then uploaded to the behavioural coding software BORIS 8.6.5 (Olivier Friard and Marco Gamba, Torino, Italy) [34] and the video was played repeatedly to capture fleeting movements, as well as allowing for easier and more accurate intercoder reliability. This method is particularly important for behaviours that happen retrospectively, i.e., to rewind from parting events allowing the coding of behaviours which precede them. 

#### 2.1.4. Determining Candidate Behaviours

Pilot observations of baboon self-scratching prior to parting prompted the selection of this behaviour in the present study. Such behaviour is also referenced in published work, within the broader category of “self-directed behaviours”. For example, “explosive hand contact” is a leave-taking behaviour in humans defined as “a rapid striking movement in which the hand(s) came in contact with either another part of the body (usually the thighs) or a foreign object (e.g., schoolbooks)- usually a slapping or striking motion” [35]. As a common behaviour in baboons, self-scratching is not unique to separation or anxiety contexts. However, investigating an increased likelihood before separation could suggest similarity with human self-directed leave takings. In this case, self-scratching could reflect anxiety around separation or the decision to separate, although testing such causation is beyond the scope of the present study.

Further to this, previous leave-taking studies suggest the importance of changing orientation (e.g., [11]) which Knapp [34] includes as part of the behaviour of “major trunk movement” and “positioning”. Visual orientation in the direction of parting [13], such as “breaking eye contact” to look away [34], is also a potential parallel across species. Orientating behaviours could be important in leave taking as they may represent a subtler intention to depart than simply moving away, as a gradual shift to the departure [11]. This contrasts with orientation towards each other which usually occurs during interaction [36], signalling the opening and closing of the dyad. In infants, turning away has been argued to represent the threat of breaking off contact [37] suggesting that it can be used intentionally to manage the ends of interactions.

Similarly, breaking eye contact or gazing away from the conspecific could act as an intermediary state where the dyad is no longer completely engaged but before final departure [6]. Similar to the example above, maintaining eye contact represents an ongoing social interaction as the channels for communication are open, whereas “turning the shoulder or refusing to make visual contact” [38] is an aggression-blocking behaviour, threatening to break off contact. 

Specific, measurable, and varied candidate behaviours must be carefully chosen and justified in order to address the methodological issue of ensuring relevant behaviours are captured. 

#### 2.1.5. Control Conditions

Combining the efforts of previous studies, we included length of interaction as a control variable. We did so by including the number of seconds from the start of the social behaviour (or activity in the case of solo condition), until the move away, as a factor in the models. This allowed us to control for the fact that restlessness could lead to some behaviours disproportionately occurring in long interactions [11]. We also included the parting type as a key variable in the model: whether the individual was parting from a social activity, leaving a solo activity, or leaving a solo activity in close proximity to another individual (see Table 1). We categorised these conditions based on whether activities were a social activity (grooming, playing, copulating) and whether they were in close proximity (estimated 1m) to another individual. This is important in our new framework because it helps us understand whether behaviour is happening significantly more at the endings of social interactions than asocial activity endings, i.e., whether such behaviour is really socially charged. 

### 2.2. Analyses

All analyses were conducted in R version 3.6.3 (R Core Team, Vienna, Austria). Analyses were conducted at the observation level, where each observation contributed one data point. Each observation had all independent variables associated with it (parting type, individuals in dyad, and duration of interaction). The dependent variables for each observation were measured in the three-minute preparting window and included the presence or absence and the frequency of each candidate behaviour. We predicted, based on the social definition of leave taking [1], that leave taking would be significantly more likely in social separation, compared to proximity separation or solo departure. 

A multilevel approach was applied to account for the fact that repeated individuals are used across observations in order to avoid pseudoreplication, i.e., each individual contributes to more than one observation and, therefore, more than one data point. This approach is ideal for a situation of repeated individuals and multiple levels, avoiding the atomistic and ecological fallacies [39] by modelling individuals and their contexts simultaneously. It also allows for missing data and the imbalance of the model, while accounting for individual differences [40]. Ultimately, this allowed an understanding of whether the current framework for measuring leave taking is both appropriate and useful. 

The analysis took a multivariate approach. As leave taking had never previously been studied in this context, we began with a descriptive investigation of what behaviours occur in the preparting window. Although these behaviours are defined and measured as distinct occurrences, it is possible that there is correlation between occurrences of different behaviours, e.g., individuals that exhibit self-scratching might also be more likely to show other behaviours such as directional eye gaze or weight shift. In this sense, we cannot treat these independent variables as truly independent of one another, and, therefore, we must use a multivariate approach. These outcomes were nested within each observation, and the structure of the model allowed both the relationship of the independent variables to be assessed, as well as the effects of other factors on these variables. 

“Choice” of behaviour can be modelled in two ways: multinomial and multivariate. Multinomial models assume the independence of irrelevant alternatives, i.e., the choices are mutually exclusive. Behavioural choices, such as those in the current study, are not mutually exclusive and may relate to more than one correlated behaviour at once. For example, in each observation, self-scratch and weight shift are not mutually exclusive; both are possible to co-occur. Therefore, we used a multivariate model, allowing for possible contemporaneous correlation in the three candidate leave-taking behaviours. 

This study took two approaches: binary leave-taking presence/absence and frequency count of the behaviour in the preparting window. Thus, we needed a model that could account for multivariate binary and count outcomes. There were two types of correlation that we needed to be aware of: correlation within individuals and correlation between possible outcomes. The General Linear Mixed Models (GLMMs) generally work well for the clustered or multilevel aspect of data analysis, but can also be extended to allow for multivariate comparisons. This is performed by creating multiple GLMMs for each response variable, and combining the responses in a single model by imposing a joint multivariate normal distribution for the variable-specific random effects. A multivariate probit model estimates several correlated binary outcomes jointly, and the Poisson version of this model can do the same for count (frequency) data. The package McGLM provides a general statistical modelling framework for normal and non-normal multivariate data analysis, designed to handle multivariate response variables. Thus, the predictions were that the candidate leave-taking behaviours would be significantly more likfmczffely to be present (binary model) and greater in frequency (frequency model) in the social condition, than the proximity or solo conditions.

### 2.3. Study Site, Subjects, and Protocol

#### 2.3.1. Gorongosa National Park

Videos were taken for this study across three months of field work (August, October, and November of 2018) in Chitengo camp, Gorongosa National Park. The first month was to test recording protocols and collect ad libitum data [41]. Following this, daily following of the baboons was conducted and videos taken during the latter two months, totalling over 65 hours of footage. This was carried out opportunistically (albeit trying to randomize across subgroups) while following to capture as many interactions as possible, rather than filming individual focal dyads. 

The park itself covers 4067 m^2^ across central Mozambique, where the base camp, Chitengo, is the home range of two troops. One of these troops, comprising of 37 individuals (Table 2), is the focus of this study, chosen due to previous identification of these individuals. Within this troop, 15 juveniles were excluded, leading to a sample of 22 adult individuals in the present study. Infants were also excluded due to a lack of comparable separation with the adult counterparts. Although future studies would benefit from valuable data on infant and juvenile separations, lack of identification at the present time prevents the control of individual replication. Additionally, we would benefit from first establishing an understanding of adult baboon leave taking, before expanding this to investigate the developmental process of the behaviour. 

Importantly, baboons of Gorongosa National Park experience a mosaic of habitats including closed-canopy savannah, rainforest, montane grasslands, rivers, and caves [42,43,44,45]. Modern environments analogous to the ones inhabited in the past by Australopithecines 4.2 to 2.3 million years ago “certainly included present day Gorongosa National Park” [46]. The camp is a tourist camp with cabins, a campsite, a restaurant, and safaris and, thus, the population is well-habituated to observer presence at close range. On the other hand, this means that the behaviour of these animals is contextualised in an anthropogenic environment, which could lead to some idiosyncrasies that are not necessarily representative of the species. Despite this, the open terrain and well-habituated troop make this setting and population ideal for recording everyday interactions of baboons. 

#### 2.3.2. Data Collection Protocol

**Figure 2:**
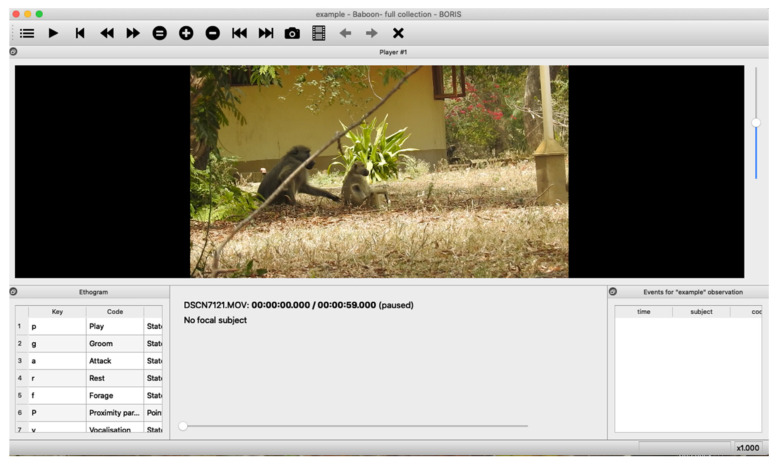
Screenshot of BORIS software.

Open access event-logging software, BORIS, was used to collect behavioural data from digitally imported videos [34] (Figure 2). This gave the advantage of rewinding from separation to record prior behaviour, something that is almost impossible to collect in real time. The unit of measurement for this study was “observations”, which was defined as being from the start of an activity (solo or joint) to the moment where the departure (solo or joint) occurs. Dyads were chosen opportunistically and exhaustively throughout the video footage, and the parting individual was defined as the first to leave the dyad (or the only individual in the case of solo activity). For each observation, the unique video ID was recorded and the observations numbered. 

Over 500 dyad observations were initially identified from the 65 hours of video footage. This number is relatively low due to excluding nonadult and unidentifiable dyads. Additionally, low visibility impacted the final number of analysed observations. Those including behaviours that could not be fully coded were removed, such as observations where foliage or other individuals obscured view. Furthermore, some observations had no clear separation, for example, if the camera cut out or vision became impaired by another individual. Observations in which the ending was caused by the joining of the third individual (or a second in the case of solo observations) were also excluded. Finally, observations that were interrupted or where the parting individual was followed were removed, as they did not meet the criteria for intentional separation. Thus, the sample of fully coded behaviours was reduced to 204. Although this sample was small, we were able to control for interindividual variation and test the presence of candidate leave-taking behaviours in social interaction endings. 

## 3. Results

These observations fall across the three categories outlined above: solo, proximity, and social. The numbers of observations in each category can be seen below in Table 3. The variation in number between the three types of separation is controlled for in the analysis. 

**Figure 3:**
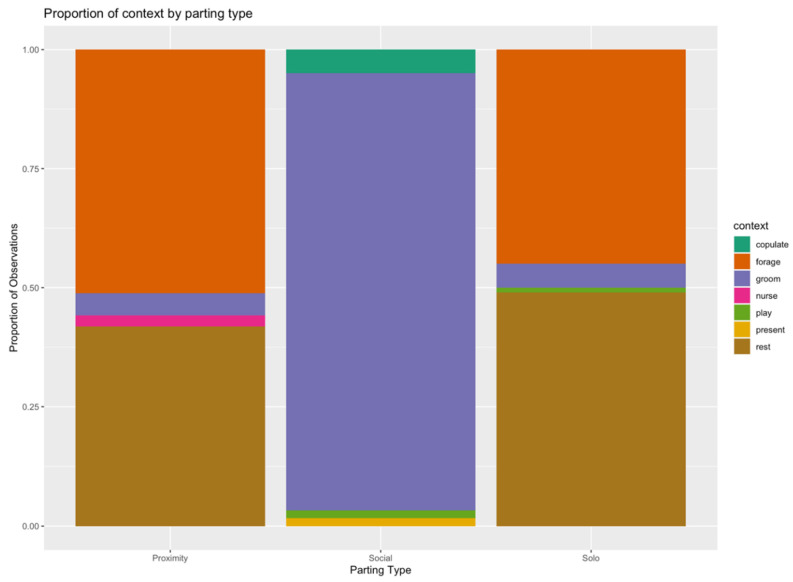
Proportion of context by parting type.

The context of observations was limited; the majority included contexts of rest, foraging, and grooming. However, as context correlated so strongly with parting type (see Figure 3), only parting type was used in the present analysis, due to the focus on the social driver of leave taking. 

There was also variation in the number of observations per individual, likely due to the variation in habituation. In the final sample of observations, only 22 individuals were identified out of the possible 37 due to the exclusion of juveniles. Furthermore, adult females Zoe (n = 3) and Maddie (n = 3) were observed much less frequently. Others, such as adult male Arrow (n = 16), were over-represented in the sample. Individual ID was included in the models to account for this. 

**Figure 4:**
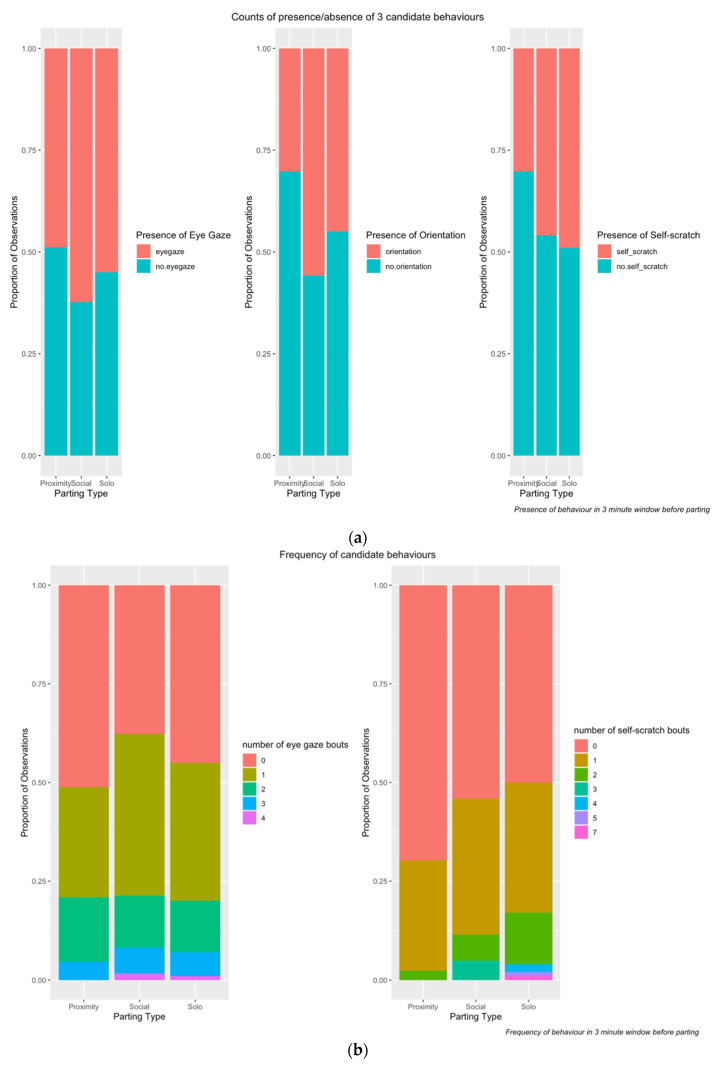
(**a**): Proportion of observations where candidate behaviours were present/absent. (**b**): Proportion of observations with each count of candidate behaviours.

Figure 4a,b illustrate the exploratory analysis of the data. Figure 4a shows the proportion of total observations (n = 204) where each of the three candidate behaviours was present and absent. Similarly, Figure 4b shows the frequency of each candidate behaviour across total observations (n = 204). These two outputs (presence/absence and frequency) reflect the basis for the two strands of investigation. 

### 3.1. IO Reliability

We assessed interobserver reliability with a second person who was naïve to the hypotheses coding over 10% of the observations. The observations used in interobserver reliability were taken at regular intervals throughout the full dataset to ensure a true cross-section of the data was compared. We considered our data to be reliably collected if the proportions of agreement between two observers using Cohen’s kappa were significantly different from those expected by chance. Cohen’s kappa values (CK) are considered fair if ranging from 0.4 to 0.6 and good if between 0.6 and 0.8 [47]. Interobserver reliability was deemed good; all measures produced similarity that was significantly more than would be expected by chance (presence of self-scratch, CK = 0.741, *p* ≤ 0.000; frequency of self-scratch, CK = 0.652, *p* ≤ 0.000; presence of gaze, CK = 0.502, *p* = 0.004; frequency of gaze, CK = 0.551, *p* ≤ 0.000; and orientation, CK = 0.584, *p* = 0.001). 

### 3.2. Statistical Analysis

We used focal sampling to record the possible leave taking of chacma baboons, investigating which of the candidate behaviours occurred, and how often, in the three minutes before parting. We analysed 205 observations in 22 individuals across solo, proximity, and social endings. We predicted, based on the social definition of leave taking [1], that behaviours would be significantly more likely in social separation compared to proximity separation or solo departure.

Self-scratching was produced in 90 observations, eye gaze in the direction of parting in 114 observations, and orientation towards parting direction in 92 observations. Observations had a mean duration of 163.66 seconds and a range of 5.05–913.86 s. 

We calculated Cook’s distances to look for influential observations that could affect the models [48]. We identified around six potentially influential observations for self-scratch, four for eye gaze, and six for orientation that could be considered outliers; however, we decided not to remove them as this variation could reflect true differences in the frequency of behaviour. Thus, we decided to run and interpret all observations to obtain first insights into leave-taking behaviour that is, as of yet, unstudied in its own right in wild nonhuman species. 

Multivariate response variable regression models allow multiple response variables to be included within one model (while holding them constant) instead of conducting several models separately, and they estimate the correlation between each response variable [49]. The models included the duration of interaction and parting type as covariates. Duration was a continuous predictor covariate and parting type had categories of social, proximity, and solo context. We included individual ID for the leaving and remaining individual as random effects to account for the repeated measures and avoid pseudoreplication. We ran two multivariate models. The first had the three response variables measured as binary outcomes (0 = behaviour absent, 1 = behaviour present, in the three-minute window prior to parting) for self-scratch, eye gaze in the direction of parting, and orientation in the direction of parting, and are referred to hereafter as the “binary” models. The second had eye gaze in the direction of parting and self-scratch measured as counts, i.e., frequency of occurrences in the three-minute window prior to parting, together with the original binary measure for orientation, and are referred to hereafter as the “frequency” models. 

A combination of logit and gamma-log error distributions was used in the models depending on the residual normality of each response variable. We used pseudo-Akaike’s information criterion (pAIC) values calculated in ‘mcglm’ to determine model selection [49]. The pAIC is similar to Akaike’s information criterion (AIC) used in model selection but contains penalty terms to account for multiple response variables in the model.

**Figure 5:**
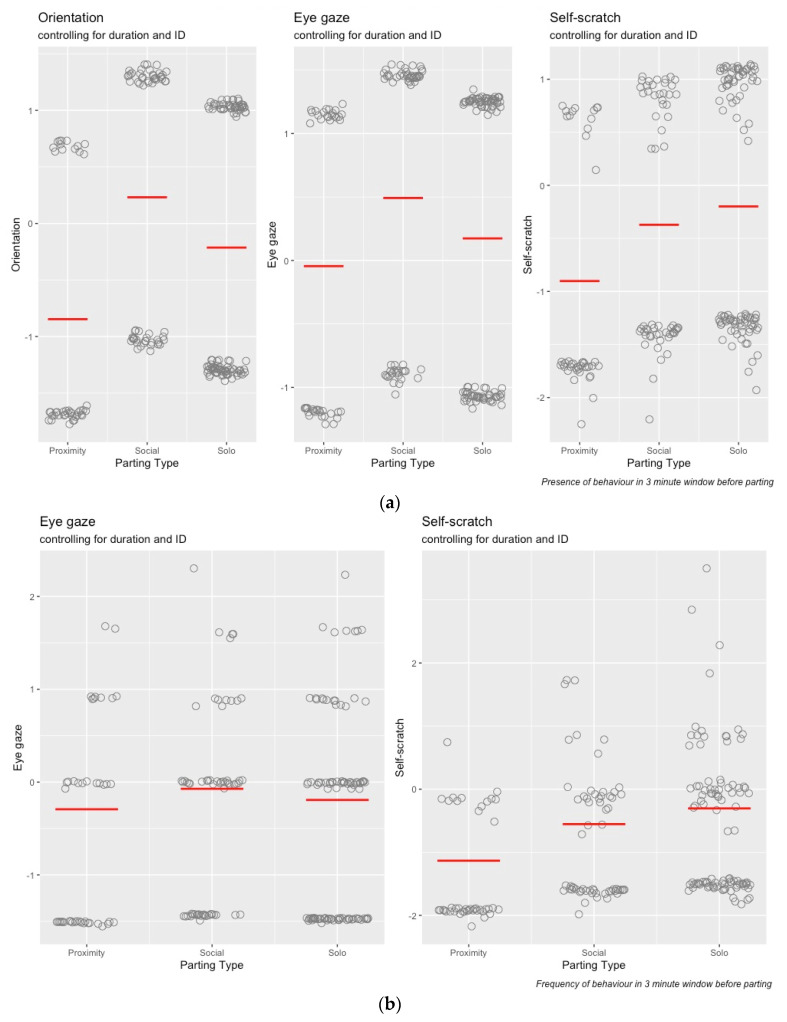
(**a**,**b**) Relationship between candidate behaviour presence and parting type.

Figure 5a,b were created using the package “visreg” [50] and illustrate the conditional relationship between the candidate behaviour and parting type. They take account of the other factors of interest, specifically, duration of interaction and individual ID. The fitted model was used to predict values of the response variable (depicted as grey circles), across the range of the chosen explanatory variable (x axis), along with the regression line. The other variables were set to their median value (for numeric variables) or most frequent category (for categorical variables). Thus, for Figure 5a, we can see that controlling for ID and duration, there were differences in the presence of candidate behaviours across the parting types. Similarly, for Figure 5b, we can see that there was some difference in frequency of self-scratch across parting type, controlling for ID and duration, whereas there was little difference in the frequency of eye gaze in the direction of parting when controlling for the same factors. The following models in the next section will investigate whether these apparent differences are statistically significant. 

After residual analysis and diagnosis (see Tables S1 and S2, and Figures S1–S4), the interpretations of the models are presented. We predicted, based on the social definition of leave taking [1], that behaviour is significantly more likely in social separation, compared to proximity separation or solo departure. Table 4, Table 5, Table 6 and Table 7 present the estimates of the regression parameters by the models, where 4 relates to the binary model (a for orientation, b for eye gaze and c for self-scratch) and 5 the frequency model (a for eye gaze and b for self-scratch). The tables show the regression parameter estimates, standard errors (SE), odds ratio (OR) with 95% CI, and z-statistics and *p* values. Table 4 illustrates that social parting type is significantly associated with orientation. Table 5 illustrates that presence of orientation is associated with social parting type, and frequency of self-scratch is associated with solo parting type.

## 4. Discussion

### 4.1. Evolutionary Context

This was the first time that control conditions were used to investigate the presence of leave taking in a wild nonhuman primate: here, the chacma baboon (*Papio ursinus*). We showed that, through controlling for leaving close proximity and solo activities, it is possible to measure leave taking in the wild. This is the first indication of leave taking, as a measure of ending social behaviours [1], existing beyond humans. 

Our results support the significance of shifting orientation to the direction of parting in the three-minute window before social separation. Increased frequency of self-scratch was associated more strongly with the solo condition, which could be due to increased anxiety of staying with the group, moving away alone, or beginning a social interaction. 

This provides preliminary evidence that social separation in baboons drives behaviour, specifically orientating in the direction of parting, and opens up a number of research hypotheses about the function of leave taking across species, such as managing separation [11], increasing the affiliative nature of interactions [51], or mitigating risky ends to interactions [52]. The results also extend Lorenz’s idea of “intention movements” [53] (p. 256), where initiating behaviours are understood by conspecifics. The current study distinguishes itself from Lorenz’s framework by eliminating instances where the behaviour is “imitated” or “contagious”, i.e., where the conspecific would also leave. The current study is concerned only with those instances where separation of dyads occurs, not “synchronised, collective cohesion” [1] (p. 10).

The presence of leave taking in baboons could suggest a deep evolutionary history of the behaviour, present since the last common ancestors of humans and baboons. Further investigation would be needed to determine the presence of leave taking in other baboon species and in more closely related species to humans such as chimpanzees or other great apes. Compared to a lack of association with orientation in the direction of parting in solo and proximity separations, this suggests that the behaviour plays a uniquely social role that could have evolved for a social function. This paves the way for understanding how leave taking functions in natural, spontaneous interactions; for example, it may assist in strengthening social bonds [51] or in minimising aggression [54]. 

### 4.2. Towards a New Framework for Leave Taking

#### 4.2.1. Definition

This study provides evidence that a social definition [1] is a useful way of determining leave taking, and that separation from close proximity is not enough to drive leave-taking behaviours. This echoes earlier assertions that leave taking is a uniquely social behaviour (e.g., [6,55,56]) and parallels assumptions about human leave taking occurring solely to manage social interaction and expectations (e.g., [6,57]). This builds upon Lockard’s previous work [11] claiming that “focused interaction is a necessary pre-condition”, from using a control of moving away together in the same direction. 

#### 4.2.2. Control Conditions

Thus, by extension, this study supports the use of definitional controls, such as separation from close proximity and solo departure, to infer the presence of leave taking before social separation. Similarly, using the length of interaction prevented behaviours that could be due to restlessness being attributed to leave taking. Such a paradigm could, and should, be applied across species and populations to further investigate the evolution of leave taking in nonhumans. 

#### 4.2.3. Levels of Separation

This also strengthens the idea of levels of separation, including the idea that leave taking could exist even in cohesive groups because of the need to successfully end social interactions [1]. This framework suggests that we need to include enquiry on different levels of separation, e.g., daily recurrent separations, and not just group-level separations such as fission or permanent separations such as group transfer. Thus, the presence of leave taking in a species such as baboons, that is typically considered cohesive [58], parallels what we see in the literature on human leave taking where both short-term, low-level separations (e.g., [11,13,59]) and long-term, high-level separations exist and are of interest [60]. 

#### 4.2.4. Data Collection Methods

This study pertains to the comment from Albert and Kessler [6] on the methodologies of leave-taking study, where they hypothesise that good research will only be possible once technology improves sufficiently to capture the fleeting nature of leave taking. This is exactly what our method achieves, both through the use of recorded high-quality video footage and the video-coding software, allowing behaviours to be retrospectively coded in minute detail at greater speed and accuracy. It means that videos can be rewound, rewatched, and slowed down to improve data validity, especially cross-observer validity. Furthermore, video archives are more accessible as they can be studied remotely now and in the future. The data collection software with a predetermined ethogram means that the same method can be applied across species and populations.

#### 4.2.5. Candidate Behaviours

Unlike most of the literature on human leave taking, this study focused only on nonvocal behaviours. Pilot observations prior to this study and indications from existing literature were able to help us identify orientation behaviour that appears to be significant prior to parting. Compared to studies such as that by Knapp et al. [35], a narrower range of candidate behaviours were used due to the application to nonhuman primates. However, it seems that there is some overlap in leave-taking behaviours across species. For example, body orientation is found in both baboons and humans, where greeting involves orienting to face one another [56] (p. 74), and leave taking involves visual-orienting signs such as turning away [13]. This is suggested in humans to signal readiness to depart, rather than simply moving away [11]. In humans, these behaviours are thought to be used to buffer verbal cues, although there is no evidence that this is the case in chacma baboons. Interestingly, conversation analysis has recently been extended to baboon behaviour with regard to orientation in greeting sequences [61]. Such studies provide support for our findings of shifting orientation as a way to break social contact and offer an exciting method to extend baboon leave-taking research. 

In summary, this study provides a definition with controls, a method, and novel candidate behaviours, which together facilitate cross-species research on leave taking. Together, this gives a clear advantage over previous definitions, not least because it can be applied across species, but also because it strengthens support for the presence of such behaviours. This allows us to further investigate the evolutionary origins of leave taking, to understand how and when it has evolved as a universal behaviour in humans. 

#### 4.2.6. Limitations

There are several limiting factors of this study, most notably that this method has thus far only been tested on a single population. It remains to be seen whether it will provide evidence for leave taking in others. Despite the sample population providing a good basis for close-range video footage and behavioural-data collection, our archive gave a limited context of behaviours, possibly affected by the anthropogenic environment. The sample size was also regrettably limited by external factors which did not allow all individuals to be identified or endings to be coded. More data collection was impossible to achieve due to the constraints posed by the COVID 19 pandemic. Automated video identification and behavioural recognition software would speed up the data collection process and account for less human error than manual coding [62].

We cannot assume that these leave-taking behaviours have been produced intentionally. Support for the social definition suggests that shifting orientation occurs significantly before ending social interactions. However, we do not yet know enough to determine whether this behaviour could constitute an intentionally produced signal of departure. One alternative explanation could be that this behaviour is more likely at the end of social interactions because of increased anxiety, in the same way predator calls were once understood [63]. Nonetheless, we cannot rule out the possibility that this is an intentionally produced gesture. Further research will test this hypothesis using previously established methods to deduce intentionality. 

## 5. Conclusions

One of the most interesting aspects of these results is that orientation in the direction of parting is more likely when leaving the social condition, as compared to the close-proximity condition, suggesting this behaviour is dependent on social interaction itself and not just the presence of other individuals. Ongoing research will look in greater detail at the dyad to determine whether this is an intentional signal of departure and whether there is a social function. Context is also important, as our results suggest that baboon leave taking is variable and not a social constraint upon leaving as in humans [56].

Interestingly, self-scratching prior to parting was significant in the solo condition. This also warrants exploration, particularly with regard to anxiety of separation from the group. Although not significant in the social condition, self-scratching did occur in many social partings, deserving further investigation as to whether there is a particular social context for its occurrence. The signal precursor route hypothesis suggests that social signals can arise from unintentionally produced asocial behaviours [11] and become specialised over time to convey information more effectively [64]. Self-directed behaviours, e.g., head scratching and beard stroking, are reported in humans as relating to uncertainty or anxiety [65], similar to their counterpart behaviours in nonhuman primates, e.g., scratching or fumbling [66]. Despite this, there is little relating these behaviours to separation specifically. Features of human cognition such as theory of mind and empathy could explain why human leave taking has become more elaborated, although potentially rooted in similar emotional, anxiety-related responses. This should be further investigated in relation to self-scratch behaviours to determine their role in solo departures. 

These results also open up the possibility of repeating the study across baboon subspecies, increasing our understanding of the cultural and ecological variation that may exist in leave taking, as it does for greeting [67]. Differences in aggressive behaviour, social cohesion, and female-bondedness may affect this propensity to leave take, and baboons would offer an ideal model species to investigate this given the radiation of related species across Africa [68]. The lack of risk in Gorongosa National Park could also impact these results: baboons there spend more time on the ground compared to other populations [45] and this could increase opportunities for such short-term, recurrent social interactions. Such opportunities increase visual proximity, and as such, leave taking may be a necessary adaptation to social life on the ground. Expanding this framework across other predominantly terrestrial species, such as chimpanzees, would help to begin exploring this idea. 

The results of this study demonstrate that leave taking may not, after all, be a result of “unique and terribly human interpersonal forces”, as claimed by Knapp [35] (p. 182). Instead, as Goffman stated, animal versions of goodbye are much harder to establish than human versions [69], but the improvements in animal behaviour research and technology have indeed made this feat possible, as predicted by Albert and Kessler [6]. Our results expand previous work on non-human leave-taking [70,71], to provide a replicable framework to compare across species. The benefits of a measurable definition, control conditions, levels of separation, digital methods, and choice of candidate behaviours demonstrate the value of this new framework for studying leave taking beyond humans. The conclusions of this study provide the first evidence of leave-taking behaviour—orientation in the direction of parting—in a wild nonhuman population. The study offers insights into the evolutionary origins of leave taking, which could have a much deeper ancestral history than previously thought [7]. The function of this behaviour and presence in other primate species will be important in determining how it has evolved to become so prominent in modern humans. 

## Figures and Tables

**Table 1:** Parting types and their definitions.

	Solo Departure	Interaction End	Proximity Departure
Individuals present	Solo individual (no individuals within 1 m)	Dyad (within 1 m)	Dyad (within 1 m)
Activity end	End solo activity, e.g., rest, forage, self-groom	End joint activity, e.g., groom, play, or copulate	End solo activity (at least one of the dyad), e.g., rest, forage, or self-groom
Movement	Moves away from current spot (>1 m)	Move away from one another (>1 m)	Move away from one another (>1 m)

**Table 2:** Summary of population demographics.

Adult Males	Adult Females	Juveniles	Troop Total
10	12	15	37

**Table 3:** Number of observations by parting type.

Parting Type	Number of Observations
Solo	100
Proximity	43
Social	61
Total	204

**Table 4:** Regression parameter estimates for joint binary model.

	Effects	Estimate	SE	z-Statistics	*p*-Value
Eye gaze	Intercept	−0.0649759376	0.3287922059	−0.1976201	0.8433423
	Duration	0.0002494702	0.0008174598	0.3051774	0.7602310
	Parting type (social)	0.5343620581	0.4121778865	1.2964355	0.1948255
	Parting type (solo)	0.2090787868	0.3727807890	0.5608626	0.5748912
Self-scratch	Intercept	−1.106534038	0.3591822027	−3.080704	0.002065119
	Duration	0.002242691	0.0008856267	2.532321	0.011331019
	Parting type (social)	0.526836372	0.4339131629	1.214152	0.224689829
	Parting type (solo)	0.697408563	0.3983954436	1.750544	0.080024571
Orientation	Intercept	0.8690858410	0.3574207691	−2.4315482	0.01503445
	Duration	0.0002375434	0.0008119219	0.2925692	0.76985144
	Parting type (social)	1.0857820340	0.4292195876	2.5296656	0.01141713 *
	Parting type (solo)	0.6328640846	0.3964570247	1.5962993	0.11042198

* denotes statistically significant values (*p*-value < 0.05).

**Table 5:** Standard errors (SE), odds ratio (OR) with 95% CI, and z-statistics and *p* values for joint binary model.

	OR	2.5%	97.5%	*p*-Value
Solo	1.000	0.999	1.002	0.760
Proximity	1.706	0.761	3.828	0.195
Social	1.233	0.594	2.559	0.575

**Table 6:** Regression parameter estimates for joint frequency model.

	Effects	Estimate	SE	z-Statistics	*p*-Value
Eye gaze	Intercept	2.87 × 10^−^^4^	0.1913975773	−1.50156733	0.1332089
	Duration	1.35× 10^−^^5^	0.0004388482	0.03064973	0.9755489
	Parting type (social)	2.12 × 10^−^^1^	0.2297794091	0.92129348	0.3568972
	Parting type (solo)	8.94 × 10^−^^2^	0.2161082851	0.41380489	0.6790170
Self-scratch	Intercept	−1.2261484383	0.3109283951	−3.943507	0.0000802985
	Duration	0.0008138371	0.0004770604	1.705942	0.0880189587
	Parting type (social)	0.5824369726	0.3587461767	1.623535	0.1044750885
	Parting type (solo)	0.8417835343	0.3312493396	2.541238	0.0110460580 *
Orientation	Intercept	0.8690858410	−0.3574207691	−2.4315482	0.01503445
	Duration	0.0002375434	0.0008119219	0.2925692	0.76985144
	Parting type (social)	1.0857820340	0.4292195876	2.5296656	0.01141713 *
	Parting type (solo)	0.6328640846	0.3964570247	1.5962993	0.11042198

* denotes statistically significant values (*p*-value < 0.05).

**Table 7:** Standard errors (SE), odds ratio (OR) with 95% CI, and z-statistics and *p* values for joint frequency model.

	OR	2.5%	97.5%	*p*-Value
Solo	1.000	0.999	1.001	0.976
Proximity	1.236	0.788	1.939	0.357
Social	1.094	0.716	1.670	0.679

## Data Availability

The data presented in this study are available on request from the corresponding author. The data are not publicly available due to further expansion of the current study.

## Supplementary Materials

The following supporting information can be downloaded at: https://www.mdpi.com/article/10.3390/ani12192577/s1, Figure S1: Pearson residuals for all regression models; Figure S2: Cook’s distance for all regression models; Figure S3: DFFITS for regression models; Figure S4: Half-normal plots for frequency models; Table S1: Pseudo-maximised log-likelihood values (plogLik), degrees of freedom (df), and pseudo-Akaike (pAIC) and Bayesian (pBIC) information criteria by structure; Table S2: Wald statistics (Wa), degrees of freedom (df), and p values for each model.
