# Supplementary material for: Yet Another Non-Unique Human Behaviour: Leave-Taking in Wild Chacma Baboons (Papio ursinus)
Source: Animals (Basel). 2022 Sep 27;12(19):2577. doi: 10.3390/ani12192577 (PMC9559683; doi:10.3390/ani12192577)
Supplement: Supplementary file 1 [file animals-12-02577-s001.zip › animals-1896079-supplementary.pdf]

animals

MDPI

Supplementary information

Table S1. Averages + standard deviations of Mandarin behaviour during different social periods.

		plogLik	Df	pAIC	pBIC
Independent binary	MV	−428.14	18	892.28	971.7812
	UV	−429.99	15	889.98	956.2310
Joint binary	MV	−427.87	21	897.74	990.4914
	UV	−429.75	18	895.50	975.0012
Independent frequency	MV	−678.77	18	1393.54	1473.0412
	UV	−694.58	16	1425.16	1491.8277
Joint frequency	MV	−678.57	21	1399.14	1491.8914
	UV	−694.54	18	1425.08	1504.5812

Table S1 shows the pseudo-log-likelihood (plogLik), degrees of freedom (df) and pseudo-Akaike (pAIC) and Bayesian (pBIC) information criteria for the proposed models. The main difference between the models is the clustering of the repeated measures (independent assumes no clustering, joint uses the repeated measure clusters). The results for table 6 show that for binary models, the pseudo-log-likelihood (pLogLik = −427.87) and pseudo-Akaike (pAIC = 897.74) and Bayseian (pBIC = 990.4914) are highest for the multivariate joint structure therefore have the best fit to the data. For the frequency model the pseudo-log-likelihood (pLogLik = −678.57) and Bayseian (pBIC = 1491.8914) are highest for the joint models compared to the independent, although the univariate and multivariate are close in values, indicating that the multivariate joint model is suitable for the data.

Table S2. Wald statistics (Wa), degrees of freedom (df) and p values for each model (*denotes statistically significant covariates).

Model	Effects	Covariate	Wald (Wa)	Df	p-value
Joint binary	Eye gaze	Duration	0.0931	1	0.7602
		Parting type	1.7964	2	0.4073
	Self-scratch	Duration	6.4126	1	0.0113*
		Parting type	3.0663	2	0.2159
	Orientation	Duration	0.0764	1	0.7823
		Parting type	6.4438	2	0.0399*
Joint frequency	Eye gaze	Duration	0.0009	1	0.9755
		Parting type	0.9330	2	0.6272
	Self-scratch	Duration	2.9102	1	0.0880
		Parting type	6.8474	2	0.0326*
	Orientation	Duration	0.0856	1	0.7699
		Parting type	6.4263	2	0.0402*

Table S2 presents the results of the Wald statistics (Wa), degrees of freedom (df) and p values for each covariate that compose the linear predictor. The results show that both covariates duration and parting type are significantly different to 0 and therefore relevant in analysis of the data. After selecting the covariate structure and analysing the effect of the covariates, we present the residual analysis and the diagnosis, to evaluate the quality of the model and investigate the presence of outliers.

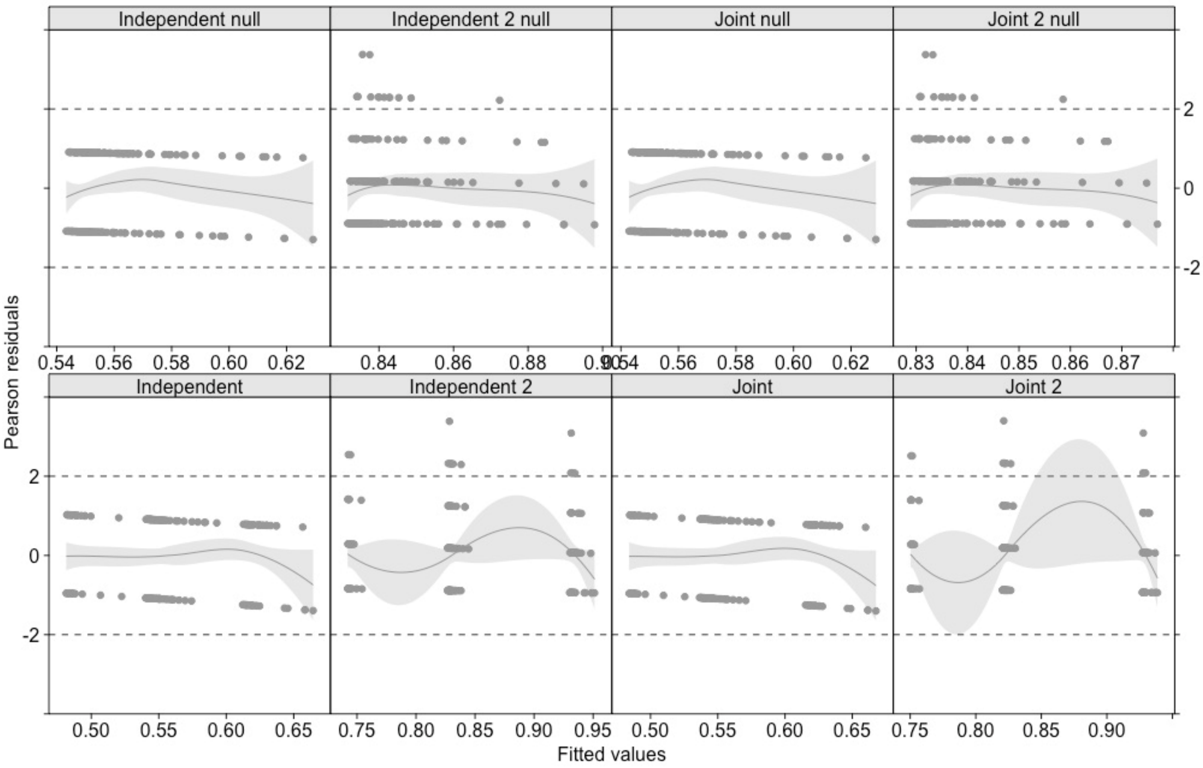

Figure S1. Pearson residuals for all regression models.

Figure S1 presents the Pearson residuals versus fitted values for the joint and independent models, for both binary (1) and frequency (2) measures. The results from figure 3 indicate that all models would be suitable as most residuals vary between -2 and +2. Although a few in the frequency model are above the upper limit, the general fit seems suitable.

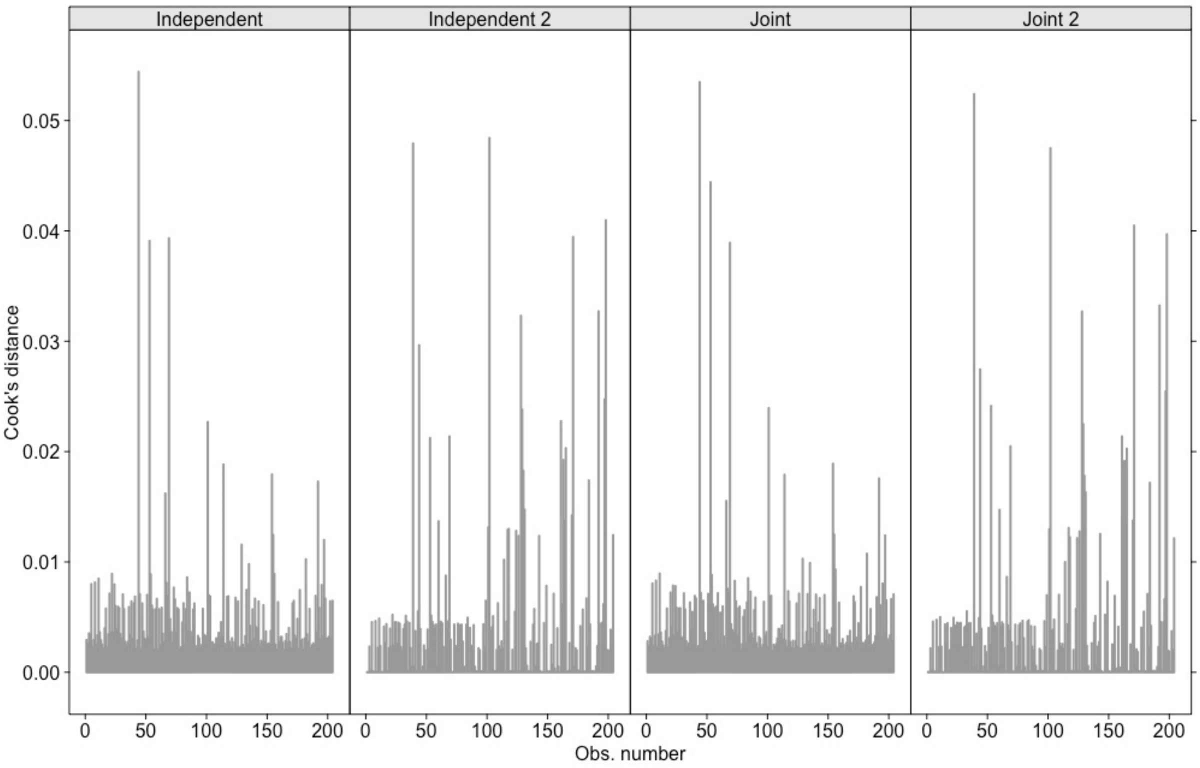

Figure S2. Cook’s distance for all regression models

Figure S2 shows the Cook’s distance versus the observation index for the proposed models. which were the influential points in the model structures, Cook 1977 – 2p/n where p is regression coefficients and n is sample size, thus values above 0.0294 (2 * 3/ 204) is considered influential. From the results of figure 8, the joint binary model only has 3 influential observations, and joint frequency ~ 6.

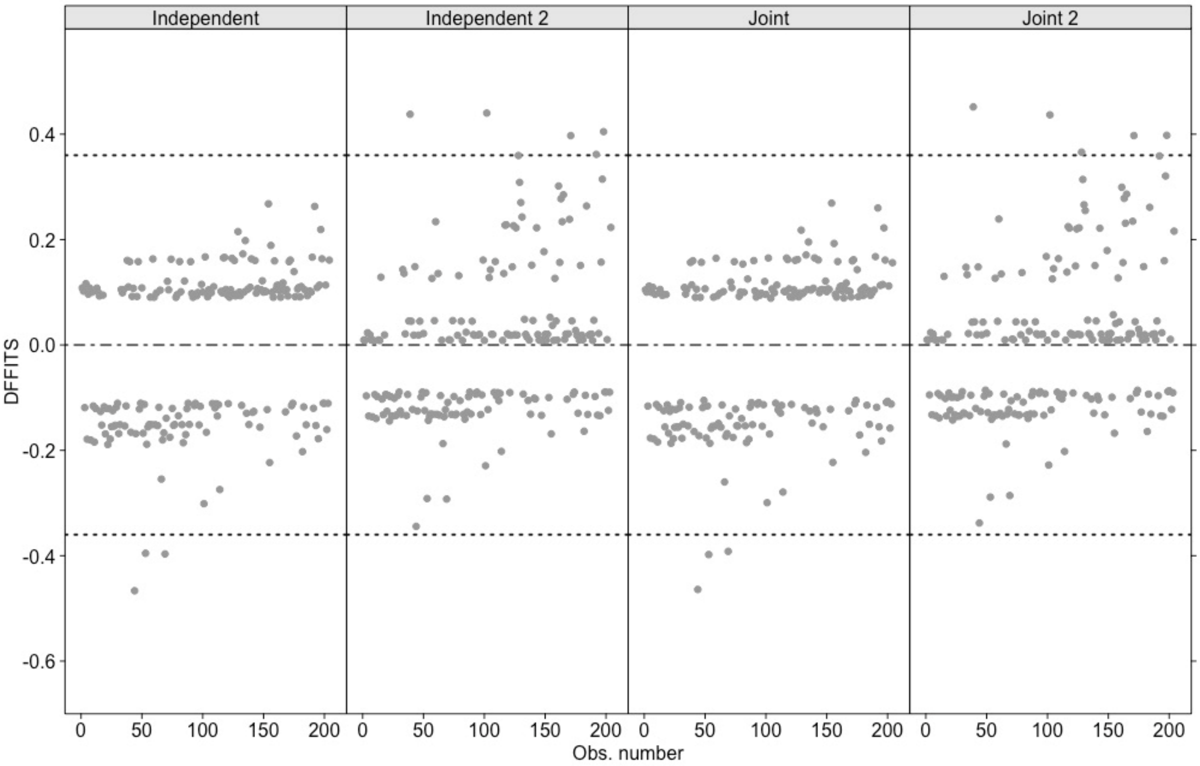

Figure S3. DFFITS for regression models.

Figure S3 shows the measure of DFFITS (Belsey et al., 1980) for the various models. This is used to measure the influence of the exclusion of the ith observation, with the cut off defined as $2\sqrt{p/n}$ where p and n refer to the number of regression coefficients and sample size, respectively. For this case the cut off will be 0.243, and although some observations lay outside of this, they are few, and so it does not render the models unsuitable.

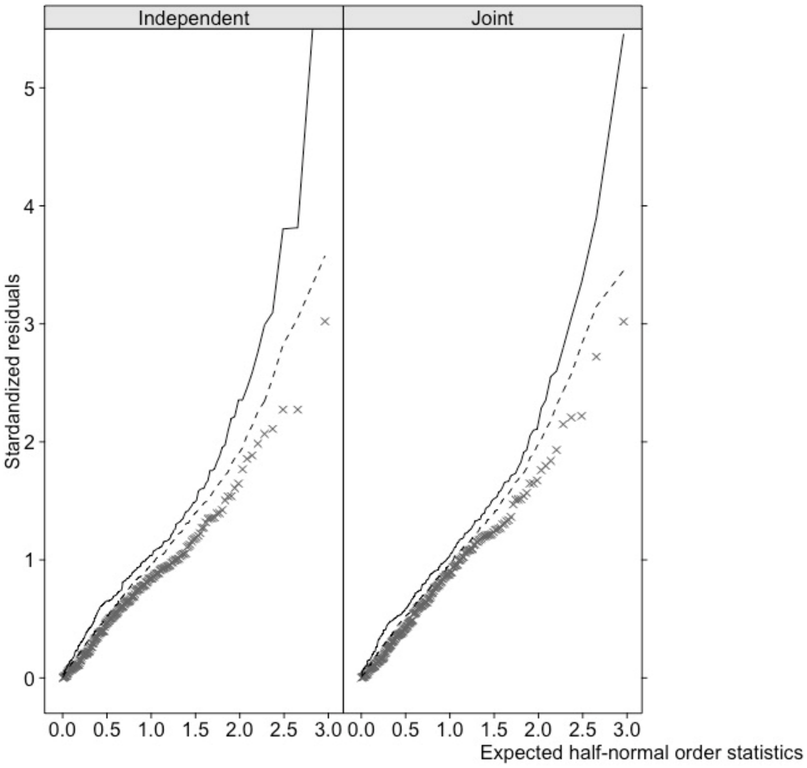

Figure S4. Half normal plots for frequency models: independent and joint .

Figure S4 shows the half-normal plots for the proposed frequency models. This technique is commonly used to evaluate the fit of the model, as well as identify outliers of how many points are outside the confidence intervals. In this case, a few points are below the lower confidence intervals, so it is assumed that the joint model provides a suitable approximation.
